# Imaging features of breast cancer subtypes on contrast enhanced ultrasound: a feasibility study

**DOI:** 10.3332/ecancer.2023.1619

**Published:** 2023-11-02

**Authors:** Stuti Chandola, Ekta Dhamija, Shashi B Paul, Smriti Hari, Atul Batra, Sandeep Mathur, S V S Deo

**Affiliations:** 1 Department of Radiodiagnosis and Interventional Radiology, IRCH, AIIMS, New Delhi 110029, India; 2 Department of Medical Oncology, IRCH, AIIMS, New Delhi 110029, India; 3 Department of Pathology, IRCH, AIIMS, New Delhi 110029, India; 4 Department of Surgical Oncology, IRCH, AIIMS, New Delhi 110029, India

**Keywords:** breast cancer, breast ultrasound, contrast-enhanced ultrasound, CEUS, breast cancer subtypes, luminal type, basal type, triple negative breast cancer, time to peak, mean transit time, peak enhancement, radiology-pathology correlation

## Abstract

The objective of this research was to study the contrast enhancement patterns of the different molecular subtypes of breast cancer on contrast-enhanced ultrasound (CEUS) using both qualitative and quantitative parameters. This prospective study included females with a single breast mass which was histopathologically proven carcinoma. B mode ultrasound (USG) and CEUS were performed in all patients during baseline assessment. Qualitative CEUS assessment encompassed enhancement pattern, presence of fill-in and washout. Quantitative assessment included measurement of peak enhancement, time to peak; area under the curve and mean transit time. A *p*-value < 0.05 was considered statistically significant for differentiating the subtypes.

The included thirty masses were categorised into two subtypes—triple negative breast cancer (TNBC) (36.7%) and non-TNBC (63.3%) subtypes. With B-mode USG, a statistically significant difference was observed between the two groups with respect to their shape and margins. TNBC lesions showed an oval shape, circumscribed margins and peripheral nodular enhancement on CEUS with the absence of fill-in even in the delayed phase (*p*-value – 0.04). The two subtypes did not significantly differ in terms of quantitative perfusion parameters. The various subtypes of breast cancer therefore possess distinct contrast enhancement patterns. CEUS potentially allows differentiation amongst these molecular subtypes that may aid in radiology-pathology (rad-path) correlation and follow up of the patients.

## Introduction

Breast cancer accounts for the highest percentage of cancer-related mortality in females worldwide [1]. The management is based upon clinical evaluation, imaging assessment and histopathological confirmation of malignancy. In addition to characterisation of the type of tumour, pathology has been able to identify and guide the treatment plan based on immunohistochemistry (IHC) analysis of the tumour. IHC has broadly classified breast cancer into four major molecular subtypes, as described by the St Gallen International Expert Panel, based upon the oestrogen receptor (ER), progesterone receptor (PR) and human epidermal growth factor receptor-2 (HER2Neu) status [2, 3]. The biological behaviour of these molecular types has been proposed to be influenced by their distinctive neovascularisation pattern and thus leads to diversity in survival outcomes and variation in the respective therapeutic options [4–6].

Imaging is an integral component of the management algorithm of breast cancer patients; mammogram (MG) is primary and ultrasound (USG) is the adjunct modality [7]. The American College of Radiology (ACR) has defined the parameters and descriptive features of breast pathologies using the imaging modalities including their size, location, morphology and associated features. After careful evaluation, these lesions are assigned an appropriate breast imaging reporting and data system category which further assists in planning the next step of evaluation for the patients. These categories serve as an important platform to reach concordance amongst clinicians, radiologists and pathologists. However, MG and USG can provide only the morphological features and not the internal vascular pattern, as seen in dynamic contrast-enhanced magnetic resonance imaging [7, 8]. Although MRI is the mainstay modality at present for perfusion assessment, it faces limitations in terms of nephrotoxic contrast usage, poor patient compliance and limited availability.

Contrast-enhanced ultrasound (CEUS) is a novel technique which enables simultaneous visualisation of mass on grey scale and enhancement characteristics post-contrast administration; thus depicting the perfusion kinetics and vascular distribution within the tumours. CEUS has been extensively studied in the assessment of liver lesions and response assessment especially in patients with deranged renal parameters because the USG contrast agent gets excreted through the respiratory system.

The intent of this pilot study was to illustrate the contrast enhancement patterns of the different molecular subtypes of breast cancer using both qualitative and quantitative parameters which may assist in radiology-pathology (rad-path) correlation and disease management.

## Materials and methods

This prospective study was conducted after obtaining approval from the institute ethics committee for a study period of April 2021 to February 2022. Written informed consent was obtained from all the patients after explaining the procedure in their vernacular language.

### Patient population

Females with a single tumour that was histopathologically proven invasive carcinoma with IHC results were included in the study at the time of baseline assessment. Exclusion criteria included history of cardio-pulmonary disease, prior treatment of breast cancer, prior allergic reaction to CEUS contrast and refusal to participate in the study.

### Image acquisition

Patients were examined on B-mode USG in supine position with ipsilateral arm raised over the head for accessibility of the entire breast parenchyma. The target mass was identified and evaluated using B-mode, colour Doppler and CEUS mode (also referred to as ‘cadence’) using Siemens Accuson, S 2000 (Siemens Healthineers, Erlangen, Germany) equipment. The B-mode USG was performed using a high-frequency linear array transducer and the size and location of the mass were recorded. Colour Doppler was applied using the power mode and any type of vascularity in the mass was documented.

### CEUS technique and evaluation

**Figure 1.**
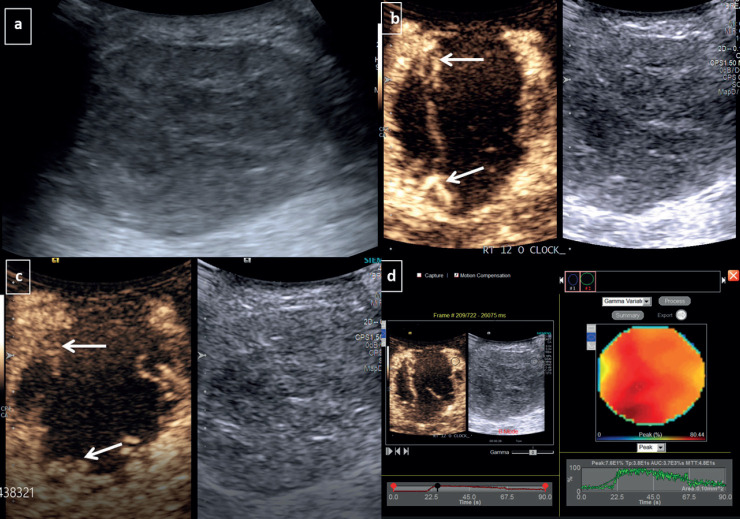
CEUS for TNBC-Grey scale USG image (a): Demonstrates a large round hypoechoic mass with circumscribed margins at 12 o’ clock position in right breast. Dual display of grey scale and CEUS image at 30 seconds (b): Shows irregular nodular rim of enhancement at periphery (arrows)- categorised as peripheral nodular pattern of enhancement. Dual image at 90 seconds (c): Shows persistent enhancing periphery without any washout or fill-in. (d): Quantitative analysis revealed that the tumour had peak enhancement (peak): 76%, TTP: 38 seconds, AUC: 3,700, MTT: 48 seconds.

The microbubble USG contrast medium used was SonoVue (Bracco, Milan, Italy) which consists of sulphur hexafluoride bubbles in a phospholipid shell. The CEUS compatible linear array transducer (9L4) was used with the breast CEUS module covering most of the tumour. The sampling frame also comprised a part of a normal breast for comparison and qualitative analysis. CEUS mode/Cadence was then turned on while applying minimal pressure on the probe and ensuring a low mechanical index activation. Once the mass was identified, a dual-mode display was put on for simultaneous demonstration of the mass in B-mode and CEUS mode (Figure 1a). Ensuring quiet breathing and minimal movement of the patient, the powdered contrast reconstituted using 5 mL normal saline was injected intravenously followed by 10 mL saline flush. The cine loop was recorded starting at the time of contrast injection which continued till pre-fixed protocol of 90 seconds. This loop was then evaluated for qualitative and quantitative analysis with the inbuilt software. Automatic motion compensating mechanisms within the software allowed reduction of the artefacts caused due to respiratory movements. Post-procedure, the patients were observed for any adverse reactions for approximately 30 minutes.

### Image analysis

The morphological description of the mass was tabulated as per the 5th edition of ACR BIRADS category- size, shape (round, oval, irregular), echogenicity (solid/cystic, hypoechoic/hyperechoic/isoechoic as compared with the fibroglandular parenchyma) and margins (angular, spiculated, microlobulated, circumscribed). The presence of vascularity on Doppler was recorded. Two radiologists (ED, SC) with 12 and 5 years of experience respectively performed and interpreted the USG and CEUS. Both were blinded to the histopathology and molecular subtype of the tumour.

Qualitative assessment of CEUS included:

- Pattern of enhancement: peripheral enhancement was defined as a smooth enhancing rim at the periphery of the lesion; peripheral nodular enhancement seen as an irregular nodular rim of enhancement at the periphery; and diffuse enhancement (enhancement of internal contents). Diffuse enhancement was further subdivided on the basis of uniformity into homogenous (uniform enhancement) and heterogeneous (enhancement with internal filling defects). The two subcategories amongst diffuse enhancement were combined together for analysis.
- Filling pattern—centripetal, i.e., enhancement of tumour periphery first with gradual central enhancement; or centrifugal (enhancement in the centre followed by peripheral tumour aspect).
- Status of washout at 90 seconds (s) – present (iso and hypoenhancing) or absent (lesion remaining hyperenhancing).

*For quantitative analysis,* the video clips were exported in digital imaging and communications in medicine format and each frame of the clip was evaluated. The frame showing the highest intralesional enhancement was then selected and a free hand region of interest (ROI) was drawn over the area of maximum enhancement taking care not to increase the size of ROI to >1 cm^2^. The image was then processed and a coloured polygonal graph depicting the degree and distribution of enhancement within the selected ROI was obtained (Figure 1b and c).

The perfusion parameters were then obtained through the inbuilt software function (Figure 1d).

The obtained perfusion parameters included-

- Peak enhancement (peak %), defined as the maximum enhancement of the area perfused in the ROI during the contrast process.
- Time to peak (TTP), defined as the time taken from contrast injection to the maximum enhancement of the ROI, which denotes the lesion’s enhancement speed.
- Area under the curve (AUC), which is the product of TTP and Peak % and is representative of the degree of perfusion of the lesion.
- Mean transit time (MTT), defined as the average time for which the agent remains within the tumour thus reflecting the washout speed of the microbubble agent.

### Histopathological evaluation

All patients underwent percutaneous biopsy and the Allred score was used for IHC evaluation- which combines the percentage of positive cells and reaction intensity to assess the ER/PR positivity [9]. A score of ≥3 (out of 8) was considered positive. For the purpose of this study; the lesions were classified as triple negative tumours (TN) – which is ER (negative), PR (negative), and Her-2 (negative); and non-TN or hormone receptor positive tumours, with either ER/PR/Her-2neu (positive).

### Statistical analysis

Data was analysed by using the statistical software Stata 14.0 (StataCorp LP, College Station, TX, USA) with biopsy results as the gold standard. The categorical variables were expressed as frequency and percentages; quantitative variable was expressed as a median. Ranksum (Mann–Whitney) test was used to compare size of the tumour between triple negative breast cancer (TNBC) versus non-TNBC groups and *chi-square*/fisher exact test was used to find the association between the two breast cancer subtypes and other clinical factors. A *p*-value <0.05 was considered statistically significant.

## Results

A total of 30 lesions were included in the study and were categorised into three major molecular subtypes: 18 cases (60%) of luminal epithelium subtype (LL), 1 case (3.3%) of Her-2 over-expression subtype (HL), and 11 cases (36.7%) with TN subtype. Amongst these, 16 cases (53.3 %) showed ER-positivity, 14 lesions (46%) were PR-positive, and 5 lesions (16.7%) showed Her-2-neu expression. For analysis, the lesions were categorised as TNBC (*n* = 11) and non-TNBC (*n* = 19) subtypes.

### Conventional USG features

No significant difference was observed amongst the TNBC and non-TNBC subtypes with respect to the lesion size (*p*-value – 0.86). While the majority were solid and irregular in shape, a statistically significant difference was observed amongst both when considering the shape and margins (*p*-value – 0.04), with TNBC presenting as well-defined oval lesions with circumscribed margins (Figure 1). TNBC showed a tendency towards solid-cystic appearance (19.2%). The majority of the lesions showed vascularity on Doppler with no significant difference amongst the two subtypes (Table 1).

### Contrast-enhanced USG

**Figure 2.**
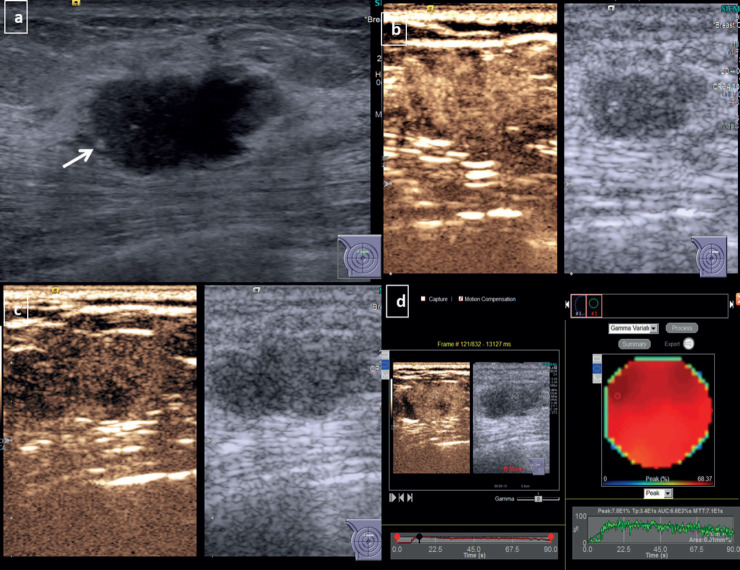
CEUS for non-TNBC subtype breast cancer-Grey scale USG image of a diagnosed HER 2 enriched subtype of breast cancer (a): Shows an irregular hypoechoic mass with angular margins and microcalcifications (arrow) at 12 o’ clock position in right breast. Dual display CEUS images at 30 seconds (b): Shows avid uniform enhancement of the entire lesion, which suggested diffuse homogeneous pattern. At 90 seconds (c): There is heterogeneous washout within the lesion. (d): Quantitative display showed peak intensity of 70%, TTP of 34 seconds, AUC of 6,600 and MTT of 71 seconds.

Non-TNBC subtype displayed a higher tendency to present with diffuse enhancement which could be heterogeneous or homogeneous (Figure 2). TNBC on the other hand, showed a distinctive tendency to display peripheral nodular enhancement (Figure 1, Table 2, *p*-value – 0.028).

For the lesions showing a diffuse variety of enhancement, all showed centripetal fill-in irrespective of subtype status. A statistically significant difference was observed with respect to the presence of fill-in; with TNBC lesions demonstrating the absence of fill-in even until the delayed phase (*p* < 0.05). The majority of the lesions (66.6%) showed washout at 90 seconds with no significant differences amongst the various molecular subtypes.

While comparing the quantitative parameters of CEUS; TNBC showed relatively longer mean MTT (53.45 seconds in TNBC versus 48.84 seconds) and TTP (38.18 seconds in TNBC versus 34.21 seconds for non-TNBC); no statistically significant difference was found amongst the two groups (Table 3).

## Discussion

Breast carcinoma is a polymorphic disease showing diversified features not only on imaging but also in terms of treatment response, progression and recurrence rates [4, 10, 11]. The advent of molecular subtyping has resulted in profound changes in treatment planning and currently IHC is essential for prognosticating this entity. The different genetic profiles influence the biological behaviour, therapeutic response and prognosis of tumour [12–14]. Breast cancer has been classified into the luminal epithelium subtype (luminal A/B), the Her-2 over-expression subtype and the TN subtype by IHC. Luminal A comprises well-differentiated tumours with low mitotic activity, highest survival rate and response to hormonal therapy against ER/PR receptors [10, 15, 9]. Luminal B tumours, on the other hand, are known to have a higher histological grade, proliferation index with variable responses to both chemotherapy and hormonal therapy [4, 16, 17]. Her-2 over-expression confers poor prognosis with a high risk of systemic metastasis [16, 17, 5], however disease-free survival has been prolonged with the advent of molecular targeted therapy [18]. The prognosis of the TN subtype remains the worst and chemotherapy forms the mainstay of treatment for this subtype [19]. This heterogeneous behaviour of breast cancer has prompted studies regarding the parameters which may potentially allow differentiation of the various subtypes from each other in recent years [13, 15, 16, 20].

Conventional USG has been used to study the various subtypes, especially TNBC subtype. TNBC tumours in our study had a propensity to show oval shape with microlobulated and circumscribed margins. This finding is in agreement with previously conducted studies which also demonstrated oval shape with circumscribed margins as a feature of TNBC rather than the luminal epithelium subtype, which are often seen as irregular mass with angular and spiculated margins consequent to slow infiltration [12, 13, 21, 22]. The smooth appearance has also been linked with the rapidity of tumour progression [15, 23].

Like any other cancer, breast cancer heavily relies upon neo-angiogenesis for its growth and metastasis and in breast cancer, the different molecular types have different patterns of angiogenesis [8, 15]. CEUS is an exclusive intravascular imaging technique that enhances the contrast between the tumour and the surrounding tissue after the injection of the contrast agent. It is readily available, easy to perform and a relatively inexpensive alternative to other modalities (MRI) used for perfusion assessment. It has been shown to overcome the limited signal-to-noise ratio and spatial resolution of Doppler USG in the evaluation of perfusion of the lesions and hence seems promising in the assessment of the perfusion characteristics of breast tumours [14, 15, 24].

The luminal epithelium subtype is the most common molecular subtype of carcinoma breast, which is also replicated in our study. The subtype presented with a diffuse enhancement pattern which could be heterogeneous or homogeneous and demonstrated a progressive centripetal fill-in. The aggressive invasive pattern and extensive stromal reaction may be a plausible cause for the association observed in our study [22, 23].

A TN subtype confers the worst prognosis to carcinoma breast with a high prevalence of early dissemination [22]. Prompt recognition of this entity has the merit of potentially allowing early institution of neoadjuvant chemotherapy. Previous studies have demonstrated no significant contrast enhancement pattern of this subtype [15]. In our study, TNBC lesions were usually found to have a peripheral nodular enhancement pattern, which consisted of nodular enhancement at the edge of the lesion with a persistent central non-enhancing area. It was subsequently observed in our study that recognition of this enhancement pattern could potentially allow detection and differentiation from the non-TNBC subtypes (*p* value < 0.05).

The majority of the lesions in our study demonstrated washout in the delayed phase, however, no statistically significant difference was observed between the two subtypes with respect to recognition of washout.

Previous studies have demonstrated significant differences in quantitative parameters in differentiating the various molecular subtypes [14, 15]. Contrary to these, we have observed no significant difference amongst the subtypes when using the quantitative contrast enhancement parameters (peak%, TTP, MTT and AUC) to differentiate TNBC and non-TNBC subtypes. A small sample size along with different measurement techniques may be the reason for this non-corroborative observation.

Although this pilot study could demonstrate the potential of CEUS in differentiating various molecular subtypes of breast cancer; it is limited by a small sample size. The results can assist in radiology-pathology (rad-path) correlation but cannot replace histopathological examination; however, the changes in CEUS parameters may aid in determining response to chemotherapy during the treatment course. A multicentric study with a verifying model is therefore warranted for validation of the results obtained in our study. Furthermore, operator subjectivity could not be completely circumvented despite all procedures being performed by the same radiologists.

## Conclusion

The various molecular subtypes of breast cancer possess distinct contrast enhancement patterns. CEUS potentially allows differentiation amongst these molecular subtypes thereby aiding in rad-path correlation; patient-centric management, stratification and treatment planning.

## Conflicts of interests

All authors declare that no conflicts exist.

## Funding

This study received an intramural institution grant for USG contrast media used within the study.

## Figures and Tables

**Table 1.** Morphological assessment of various subtypes of breast cancer.

Subtypes	Mean size (cm)	Shape		Echogenicity		Margins				Vascularity on Doppler	
		Irregular	Oval	Solid- hypoechoic	Solid-cystic	Microlob-ulated	Spiculated	Angular	Circums-cribed	*p*	*A*
Non-TNBC (*n* = 19)	4.3 ± 1.55	19 (100%)	0 (0)	19 (100%)	0 (0)	6 (31.6%)	10 (52.6%)	3 (15.8%)	0 (0)	17 (89.5%)	2 (10.5%)
TNBC (*n* = 11)	4.34 ± 2.88	8 (72.7%)	3 (27.3%)	9 (81.8%)	2 (19.2%)	4 (36.4%)	2 (18.2%)	2 (18.2%)	3 (27.2%)	9 (81.8%)	2 (19.2%)
*p* value		**0.04**		0.12		*0.041				0.0	

Bold values indicate significant association

* Indicates *p* value <0.05 of circumscribed versus non circumscribed margins in differentiating TNBC versus non-TNBC subtype

**Table 2.** Qualitative contrast enhancement parameters of the different molecular types of breast cancer.

Subtypes	Enhancement pattern				Filling pattern			Washout status at 90s	
	Peripheral	Peripheral nodular	Diffuse-homogenous	Diffuse-heterogeneous	Centripetal	Centrifugal	Nil	Present (iso/hypoenhancement at 90s)	Absent (hyperenhancement)
Non-TNBC (*n* = 19)	1 (%)	2 (%)	8 (%)	8 (%)	16 (%)	0 (0)	3 ()	12 ()	6 ()
TNBC (*n* = 11)	0 (0%)	6 (54.5%)	3 (27.2%)	2 (18.2%)	5 (45.4 %)	0 (0%)	6 (54.6%)	8 (72.7%)	3 (27.3%)
Total	1	8	11	10	21	0	9	20	10
*p* value	^*^0.028				^**^0.042			1	

Bold values indicate significant association

* Indicates *p* value <0.05 of diffuse versus peripheral nodular enhancement in differentiating TNBC versus non-TNBC subtype

** Indicates *p* value <0.05 of presence of fill-in when differentiating TNBC versus non-TNBC subtype

**Table 3.** Mean quantitative CEUS perfusion parameters of the different molecular types of breast cancer.

Subtype	PEAK (%)	TTP (seconds)	AUC (% seconds)	MTT (seconds)
Non-TNBC (*N*–19)	61.35 ± 14.5	34.21 ± 11.18	4,900 ± 2,796.4	48.84 ± 27.73
TNBC (*n*–11)	65.09 ± 11.54	38.18 ± 14.48	4,027.27 ± 1,928.25	53.45 ± 20.64
*p* value	0.23	0.2	0.18	0.31
